# C-853-01. Anti-Xa–Guided LMWH Prophylaxis in Adult Burn Patients: A Systematic Review of Current Evidence

**DOI:** 10.1093/jbcr/irag033.069

**Published:** 2026-04-06

**Authors:** Siam K Rezwan, Aman F Tahir, Kosuke Kawai, Scott B Hu, Andrew J Vardanian, Maryum H Merchant

**Affiliations:** David Geffen School of Medicine at University of California Los Angeles, Los Angeles, CA; David Geffen School of Medicine at University of California Los Angeles, Los Angeles, CA; University of California Los Angeles, Los Angeles, CA; Department of Pulmonary/Critical Care, University of California Los Angeles, Los Angeles, CA; Division of Plastic and Reconstructive Surgery, Keck School of Medicine, University of Southern California, Los Angeles, CA; Department of Pulmonary/Critical Care, University of California Los Angeles, Los Angeles, CA

## Abstract

**Introduction:**

Burn injury creates a prothrombotic and pharmacokinetically unstable state complicating venous thromboembolism (VTE) prophylaxis. Fixed-dose low-molecular-weight heparin (LMWH) often yields subprophylactic anti-Xa levels, prompting interest in anti-Xa–guided dosing. To date, no comprehensive review has focused exclusively on anti-Xa–guided LMWH in burn patients. We synthesized evidence comparing anti-Xa–guided LMWH with fixed dosing in adult burn inpatients and outlined implications for practice and research.

**Methods:**

We systematically searched PubMed, Embase, and Cochrane with a medical librarian through September 2025. We included adult burn studies of LMWH prophylaxis comparing anti-Xa–guided adjustment with fixed dosing, reporting VTE, bleeding, anti-Xa levels, dose changes, or mortality. Two reviewers independently screened and extracted data. Heterogeneity in targets, monitoring schedules, and populations precluded pooling.

**Results:**

Seven studies spanning 2004–2025 met inclusion criteria. No randomized trials were identified; all were single-center observational designs, majority retrospective. Sample sizes ranged from small case series to cohorts exceeding 800 patients. Across studies of fixed-dose LMWH prophylaxis, a large proportion of burn patients failed to achieve prophylactic anti-Xa levels. In cohorts where anti-Xa–guided adjustment was implemented, attainment of target levels improved substantially. VTE events were reported in both strategies, but trends favored anti-Xa–guided protocols, with several studies noting fewer DVT or PE events in adjusted groups. Importantly, bleeding complications were infrequent overall and did not increase with anti-Xa monitoring or dose escalation. Mortality was variably reported and not consistently different between groups.

**Conclusions:**

Evidence, though limited and low certainty, indicates fixed-dose LMWH often leaves burn patients subtherapeutic, while anti-Xa–guided adjustment improves adequacy without added bleeding. Although observational and susceptible to bias, these findings support individualized dosing as a potential strategy to lower VTE risk, warranting prospective validation.

**Applicability of Research to Practice:**

Until definitive trials, burn centers should recognize high risk of subtherapeutic anticoagulation with fixed-dose LMWH, especially in patients with large TBSA burns, obesity, or fluid shifts. Centers with resources may adopt structured anti-Xa monitoring with standardized timing and escalation algorithms, paired with surveillance for bleeding and VTE, as an interim quality-improvement measure.

**Funding for the study:**

N/A.

